# Supplementary material for: Bicarbonate activation of the monomeric photosystem II-PsbS/Psb27 complex
Source: Plant Physiol. 2023 May 18;192(4):2656–71. doi: 10.1093/plphys/kiad275 (PMC10400029; doi:10.1093/plphys/kiad275)
Supplement: kiad275_Supplementary_Data [file kiad275_supplementary_data.pdf]

Supplemental Data for

Bicarbonate Activation of Monomeric Photosystem II-PsbS/Psb27 Complex

Andrea Fantuzzi[a1], Patrycja Haniewicz[bc1], Domenica Farci[d], M. Cecilia Loi[e], Keunha Park[a], Claudia Büchel[f], Matthias Bochtler[cg], A. William Rutherford[a*], Dario Piano[ce]

This PDF file includes:

Supplemental Text S1-S3

Supplemental Figs. S1-S4

Supplemental Table S1

References for SI

Supplemental Text S1

Comparison of the UV-vis absorption and CD spectra (Figs. 2B and 2D) in the two types of PSII monomers, showed only minimal differences limited to the Soret region of the spectra. Given the lack of carotenoids and chlorophylls in the crystal structures of PsbS (except for an adventitious chlorophyll hydrophobically bound at the interface between the two monomers) and Psb27 (Fan *et al*., 2015; Xingxing *et al*., 2018), the presence of these subunits in PSIIm-S/27 cannot be directly responsible for these spectroscopic differences. A more likely explanation is that they arise from the presence of sub-stoichiometric amounts of CP26 and/or CP29 (Fig. 2A), which were reported earlier as contaminants of PSIIm-S/27 in the mass spectrometry analysis (Haniewicz *et al*., 2013). These contaminants are also likely responsible for the greater intensity in the steady-state fluorescence emission spectrum at 675 nm (Fig. 2C) and the increased background levels of fluorescence in the kinetic experiments seen in PSIIm-S/27 (see Fig. 3A and below for more details). The increase in the fluorescence could also be due to a specific effect of PsbS and Psb27 binding, resulting in a perturbation of the fluorescence in line with literature reports of similar higher fluorescence background levels in monomeric PSII with bound Psb27 (Regel *et al*., 2001; Mamedov *et al*., 2007).

Supplemental Text S2

A large difference was observed when comparing the fluorescence kinetics experiments (Fig. 3A), where both F_0 and F_m for PSIIm-S/27 were shifted to higher values compared to PSIIm. This observation could lead to the erroneous conclusion that the presence of the additional subunits caused a decrease in the maximum quantum yield of PSII (F_v/F_m) via a non-photochemical quenching mechanism (Ruban, 2016). On the contrary, upon close examination of the data presented in figure 3, it seems clear that the two data sets have very similar amplitudes, but with the PSIIm-S/27 curve shifted to higher fluorescence intensities by the addition of a constant fluorescence background. The increased fluorescence does not therefore indicate a change in the quantum yield of charge separation. This is in line with the observed higher steady state fluorescence (Fig. 2C) and is similar to what is seen when comparing cyanobacteria and eukaryotic phototrophs. Cyanobacteria have often been considered to show lower values of Fv/Fm when compared with eukaryotic phototrophs. This has been shown to be due to the phycobilisomes, most probably disconnected from the reaction centers, leading to an anomalous increase in the fluorescence background that results in an apparent reduction

of the F_v/F_m (Ogawa and Sonoike, 2016). When this contribution is subtracted, the measured value of F_v/F_m is equivalent to that measured in eukaryotic phototrophs for fully functional PSII (Kalaji *et al.*, 2017; Santabarbara *et al.*, 2019). In a similar way, a constant fluorescence background, which is not contributing to fluorescence changes due to PSII photochemistry, shifts both F_0 and F_m in PSIIm-S/27 samples (Fig. 3A).

Supplemental Text S3.

Below is a list of examples from the literature in which small subunits of PSII appear to influence electron transfer through the quinones acceptors of PSII. Some of these might be relevant to the present report.

Mutants lacking PsbJ in tobacco showed an increased lifetime (x100) of the reduced primary quinone $Q_A^{•-}$ and damped oscillations in the flash dependence of the $S_2Q_B^{•-}$ recombination, suggesting altered Q_A to Q_B electron transfer (Regel *et al.*, 2001). The ΔpsbJ mutant showed an increased level of F_0 similar to the observation here in the PSIIm-S/27. Mutants with a disrupted psbX gene showed evidence of reduced binding or turnover of Q_B (Katoh and Ikeuchi, 2001). Mutants lacking PsbH were found to show slower kinetics of $Q_A^{•-}$ oxidation, but wild-type behaviour was recovered by addition of bicarbonate (Komenda *et al.*, 2002). Both PsbH and PsbX are located between D2 and CP47, where PsbS is suggested to bind by its interaction with CP29 (Correa-Garvis *et al.*, 2016), while PsbY and PsbJ are in proximity to cyt b_{559}, next to D2.

X-ray crystallography model of PSII in mutants lacking the PsbM, showed differences in the orientation of the bicarbonate (Uto *et al.*, 2017). Double mutants lacking both PsbM and PsbY in *Synechocystis* sp. PCC 6803, where shown to be more prone to photodamage and have slightly slower kinetics of $Q_A^{•-}$ oxidation, which were faster when bicarbonate was added (Biswas and Eaton Rye, 2018). Furthermore, mutants lacking PsbY in *Arabidopsis* were found to have slower $Q_A^{•-}$ to Q_B electron transfer with smaller amplitudes for the fast $Q_A^{•-}$ to either Q_B or $Q_B^{•-}$ and a larger amplitude for the intermediate phase attributed to the exchange of Q_BH_2 with the PQ of the pool. These PsbY mutants also lacked a thermoluminescence band for $S_2Q_B^{•-}$ recombination (Von Sydow *et al.*, 2016). The authors linked this phenomenology and the alterations in the redox state of cyt b_{559} to a role of PsbY on cyclic electron flow around PSII and regulation of photodamage (Von Sydow *et al.*, 2016).

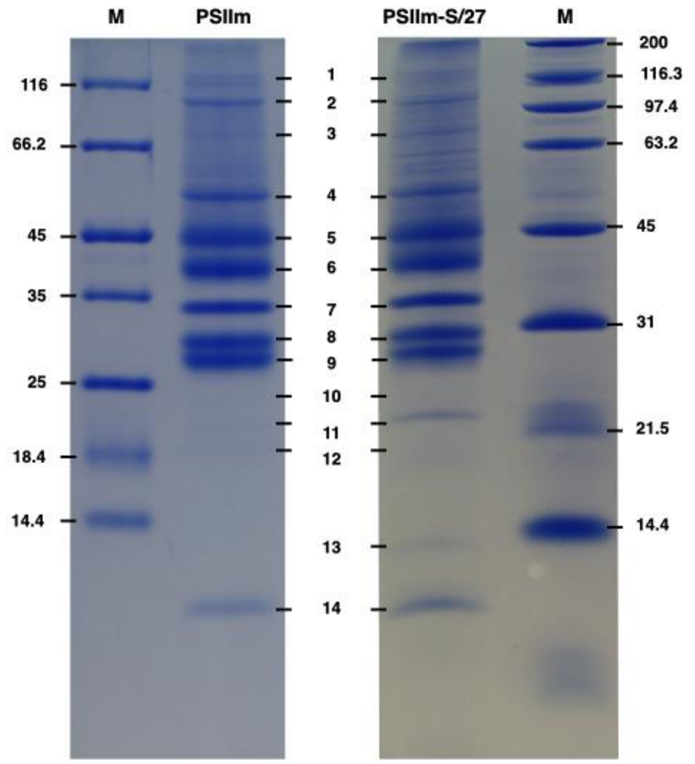

Supplemental Figure S1: Comparisons of the PSIIm and PSIIm-S/27. Coomassie Blue Stained SDS-PAGE of the PSIIm and PSIIm-S/27. These are the same SDS-PAGE shown in figure 2A, where the main bands (from 1 to 14) were subjected to a mass spectrometry analysis (Haniewicz *et al*., 2013; Haniewicz *et al*., 2015; see Table S1 for details). The bands are numbered as shown in table S1. The lanes labelled as M indicate the molecular weight markers.

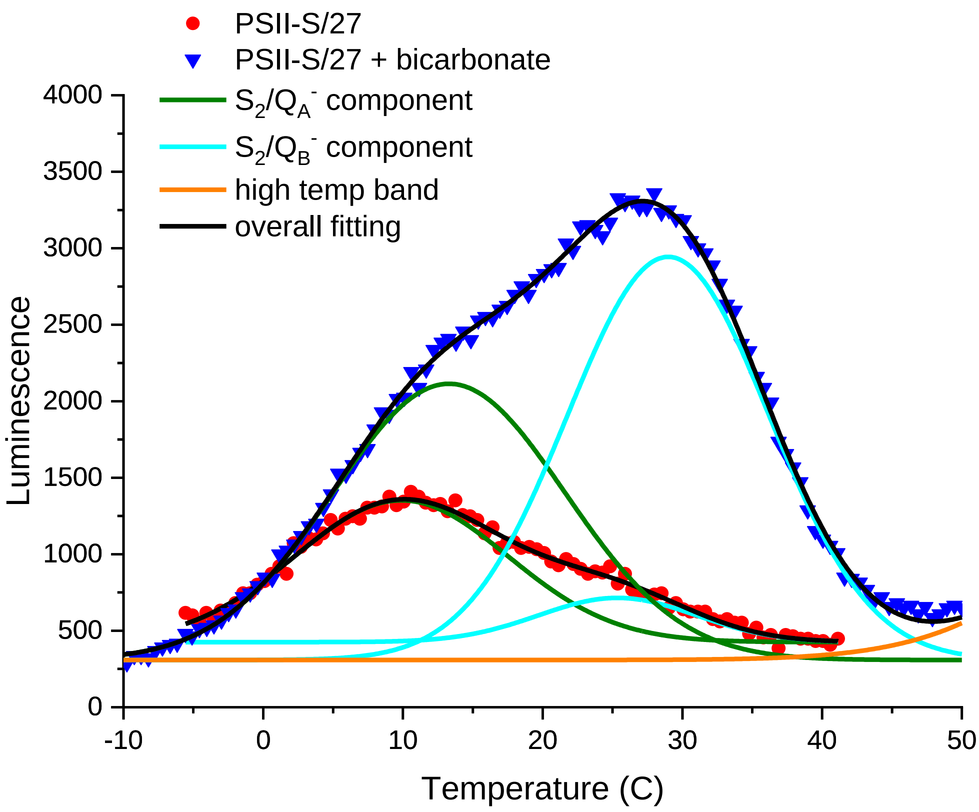

Supplemental Figure S2: Fitting of the thermoluminescence data for PSIIm-S/27 in the absence (red circles) and presence (blue triangles) of 5 mM bicarbonate. Measurements were carried out in 20 mM MES pH 6.5, 5 mM $MgCl_2$. A single saturating flash was given at 5 °C and then the sample rapidly cooled to -10 °C. Scan rate was 0.5 °C/s. Fitting was carried out in OriginLab™ using individual gaussians for each component. The black line represents the overall fit. The dark green and cyan lines represent the $S_2Q_A^{\bullet-}$ and $S_2Q_B^{\bullet-}$ respectively. The orange line represents the contribution of the high temperature band.

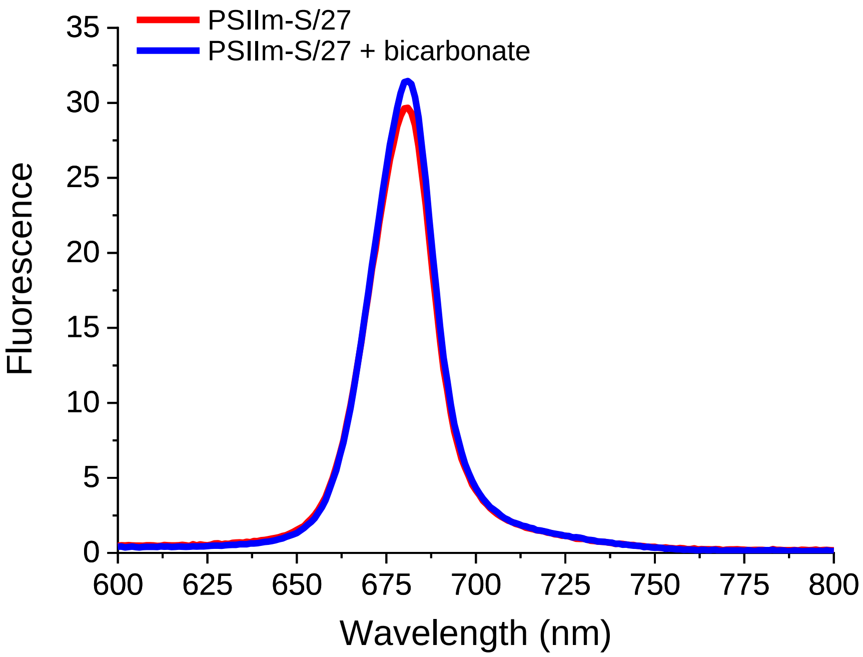

Supplemental Figure S3: Room temperature fluorescence for PSIIm-S/27 in the absence (red line) and presence (blue line) of added 5 mM bicarbonate. Measurements were carried out at 20 °C, in 20 mM MES, 5 mM $MgCl_2$, pH 6.5.

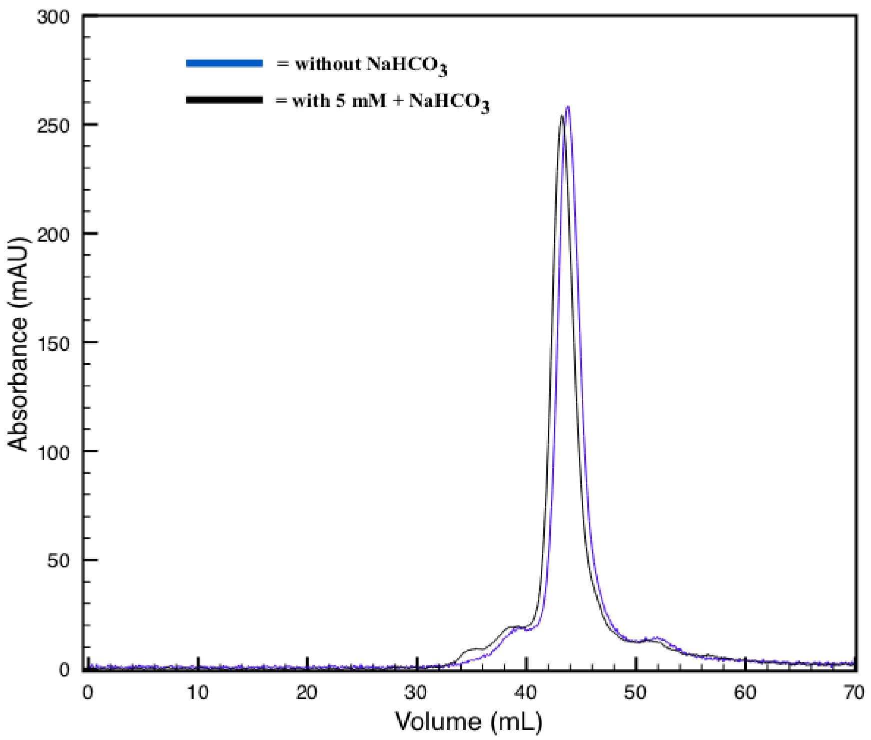

Supplemental Figure S4: The effect of bicarbonate on the retention of PsbS and Psb27 in PSIIm-S/27 samples analysed by size exclusion chromatography (SEC). SEC experiments with PSIIm-S/27 in presence (black line) and absence (blue line) of bicarbonate did not show substantial differences suggesting that the bicarbonate binding does not lead to the subunits release.

Supplemental Table S1: Mass spectrometry analysis of PSIIm and PSIIm-S/27. Based on previous results (Haniewicz *et al*., 2013; Haniewicz *et al*., 2015), a comparison of the PSII samples (Fig. 2A and S1) was done with a semi-quantitative approach. As shown, the bands with mass higher than 45 kDa are not directly linked with PSII and appear to be co-purified components. For the PSII components, the main difference between the two samples is the presence of the subunits PsbS and Psb27 in the PSIIm-S/27. We note that PSIIm also seems to lack the small subunits PsbJ, PsbQ and PsbP. We, nevertheless, consider that these differences are due to an artifactually low level of detection of these subunits in the mass spectrometry, because western blot analysis published previously on similar preparations (Haniewicz *et al.*, 2013; Haniewicz *et al.*, 2015), indicated that these subunits were present. The asterisks indicate the subunits for which western blots were performed. The semi-quantitative estimation of protein abundance was done according to the following index (spectral count/protein mass kDa), where higher values indicate a higher abundance of the protein in the sample: $i < 0.25$ (+); $0.25 < i > 0.50$ (++); $0.50 < i > 0.75$ (+++); $i > 0.75$ (++++).

Protein	Theoretical Mass (kDa)	Apparent Mass (kDa)	Band	Localization	PSIIm	PSIIm-S/27
**glycine dehydrogenase [decarboxylating]**	114.5	117	1	Mitochondrion	+	+
**glycine dehydrogenase P subunit**	110.9	110	2	Mitochondrion	+	+
**V-type proton ATPase catalytic subunit A/B**	68.8	70	3	Vacuole	+	++
**ATP synthase subunit beta**	59.8	57	4	Mitochondrion	++++	++++
**CP47**	56	45	5	Chloroplast	++++	+++*
**CP43**	50.2	40	6	Chloroplast	++++	+++*
**PsbO**	34.9	33	7	Chloroplast	+	++++*
**D2**	39.5	30	8	Chloroplast	++++	+++
**D1**	38.5	29	9	Chloroplast	++++	+++*
**PsbP**	28.6	24	10	Chloroplast	0	+*
**PsbS**	29	22	11	Chloroplast	0	++*
**PsbQ**	24.1	19	12	Chloroplast	0	+*

**Psb27**	18.7	14	13	Chloroplast	0	++
**PsbE**	9.4	10	14	Chloroplast	++++	++++*
**PsbF**	4.5	10	14	Chloroplast	+	+*
**PsbH**	8	10	14	Chloroplast	++++	++++
**PsbJ**	4.1	10	14	Chloroplast	0	+++
**PsbL**	4	10	14	Chloroplast	+	++++

References

Biswas S, and Eaton-Rye JJ (2018) PsbY is required for prevention of photodamage to Photosystem II in a PsbM-lacking mutant of *Synechocystis* sp. PCC 6803. Photosynthetica **56**: 200-209.

Correa-Galvis V, Poschmann G, Melzer M, Stühler K, Jahns P (2016) PsbS interactions involved in the activation of energy dissipation in Arabidopsis. Nat. Plants **2**:15225.

Fan M, Li M, Liu Z, Cao P, Pan X, Zhang H, Zhao X, Zhang J, Chang W (2015). Crystal structures of the PsbS protein essential for photoprotection in plants. Nat Struct Mol Biol. **22**:729-35.

Haniewicz, P, De Sanctis, D, Büchel, C, Schröder, WP, Loi, MC, Kieselbach, T, Bochtler, M, Piano, D (2013). Isolation of monomeric photosystem II that retains the subunit PsbS. Photosynth Res. **118**:199-207.

Haniewicz P, Floris D, Farci D, Kirkpatrick J, Loi MC, Büchel C, Bochtler M, Piano D (2015) Isolation of Plant Photosystem II Complexes by Fractional Solubilization. Front. Plant Sci. **6**:1100

Kalaji HM, Schansker G, Brestic M, Bussotti F, Calatayud A, Ferroni L, Goltsev V, Guidi L, Jajoo A, Li P, Losciale P, Mishra VK, Misra AN, Nebauer SG, Pancaldi S, Penella C, Pollastrini M, Suresh K, Tambussi E, Yanniccari M, Zivcak M, Cetner MD, Samborska IA, Stirbet A, Olsovska K, Kunderlikova K, Shelonzek H, Rusinowski S, Bąba W (2017) Frequently asked questions about chlorophyll fluorescence, the sequel. Photosynth Res. **132**:13-66.

Katoh H., Ikeuchi M. (2001) Targeted Disruption of psbX and Biochemical Characterization of Photosystem II Complex in the Thermophilic Cyanobacterium *Synechococcus elongatus*. Plant and Cell Physiol. **42**: 179–188

Komenda J, Lupinkova L, Kopecky J (2002) Absence of the psbH gene product destabilizes Photosystem II complex and bicarbonate binding on its acceptor side in *Synechocystis* PCC 6803. Eur. J. Biochem. **269**: 610-619.

Mamedov F, Nowaczyk MM, Thapper A, Rogner M and Styring S (2007) Functional characterization of monomeric Photosystem II core preparations from *Thermosynechococcus elongatus* with or without the Psb27 protein. Biochemistry **46**: 5542-5551.

Ogawa T. and Sonoike K (2016) Effects of Bleaching by Nitrogen Deficiency on the Quantum Yield of Photosystem II in Synechocystis sp. PCC 6803 Revealed by Chl Fluorescence Measurements. Plant Cell Physiol. **57**: 558-567.

Regel RE, Ivleva NB, Zer H, Meurer J, Shestakov SV, Herrmann RG, Pakrasi HB, Ohad I (2001) Deregulation of electron flow within Photosystem II in the absence of the PsbJ protein. The J. of Biol. Chem. **276**: 41473-41478

Ruban AV (2016) Nonphotochemical Chlorophyll Fluorescence Quenching: Mechanism and Effectiveness in Protecting Plants from Photodamage. Plant Physiol. **170**: 1903–1916.

Santabarbara S, Villafiorita Monteleone F, Remelli W, Rizzo F, Menin B, Casazza AP (2019) Comparative excitation-emission dependence of the FV/FM ratio in model green algae and cyanobacterial strains. Physiologia plantarum **166**: 351-364.

Uto S, Kawakami K, Umena Y, Iwai M, Ikeuchi M, Shen J-R, Kamiya N (2017) Mutual relationships between structural and functional changes in a PsbM-deletion mutant of photosystem II. Faraday Discuss. **198**: 107-120

Von Sydow L, Schenkert S, Meurer J, Funk C, Mamedov F, Schroder WP (2016) The PsbY protein of Arabidopsis Photosystem II is important for the redox control of cytochrome b_{559}. Biochem Bioph Acta **1857**: 1524-1533

Xingxing C, Jiuyang L, Huan Z, Fudong L, Shuya Z, Min X, Ke R, Yuhua W, Aigen F (2018) Crystal structure of Psb27 from Arabidopsis thaliana determined at a resolution of 1.85 Å. Photosynth Res. 136(2):139-146.
